# Supplementary material for: The interactome of intact mitochondria by cross-linking mass spectrometry provides evidence for coexisting respiratory supercomplexes
Source: Mol Cell Proteomics. 2017 Dec 8;17(2):216–32. doi: 10.1074/mcp.RA117.000470 (PMC5795388; doi:10.1074/mcp.RA117.000470)
Supplement: Supplemental Data [file supp_RA117.000470_133922_0_supp_23978_fzffwf.zip › spectra_annotation/mito_spectra_annotation/154-1-7-1-9-1.pdf]

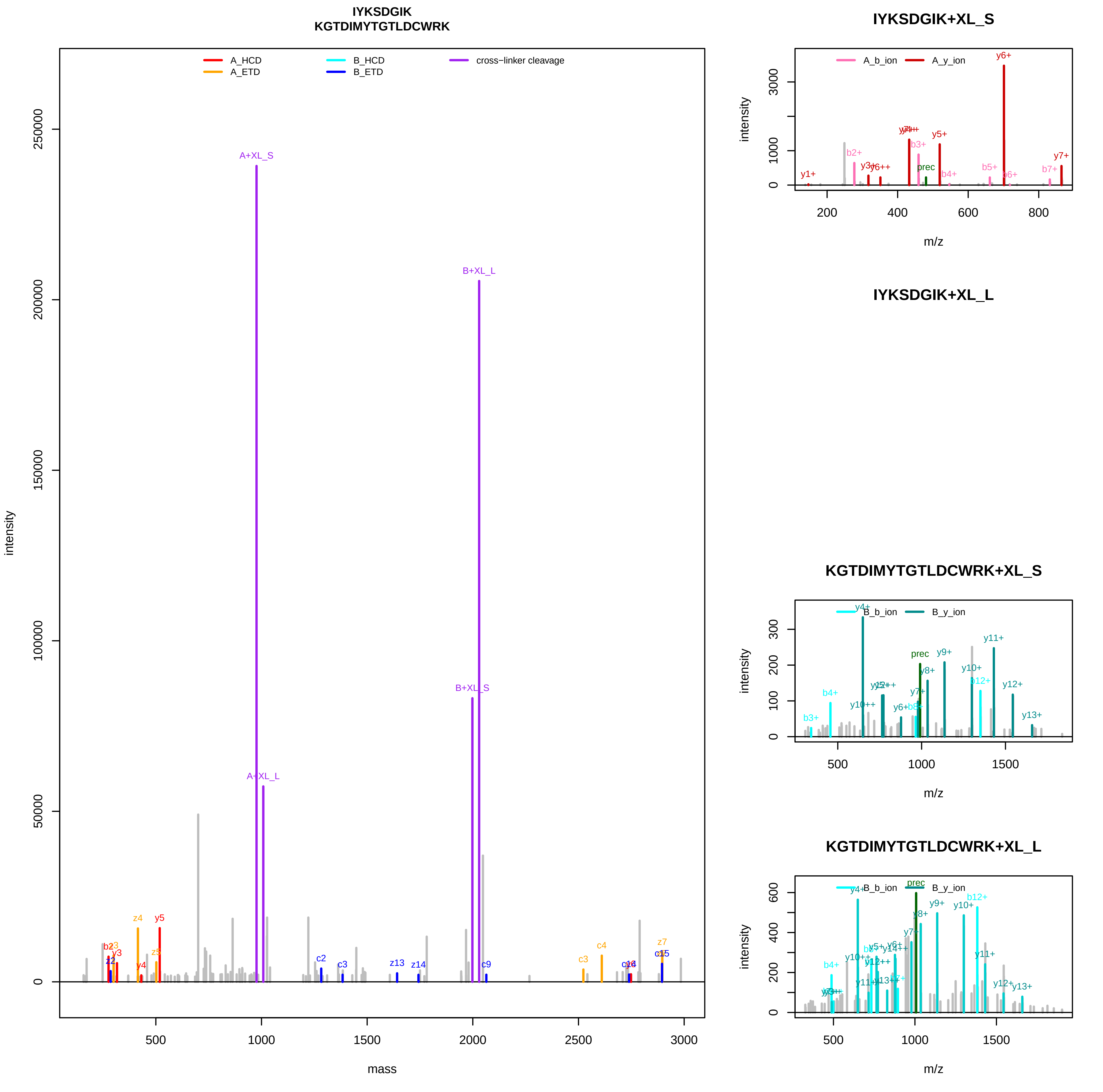
IYKSDGIK
KGTDIMYTGTLDCWRK
A_HCD
A_ETD
B_HCD
B_ETD
cross−linker cleavage
A+XL_S
B+XL_L
B+XL_S
A+XL_L
intensity
mass
0
50000
100000
150000
200000
250000
500
1000
1500
2000
2500
3000
b2
y3
z2
z4
y5
y4
c2
c3
z13
z14
c9
c3
c4
z7
c15
IYKSDGIK+XL_S
A_b_ion
A_y_ion
y1+
b2+
y3+
y6++
y4++
b3+
y5+
prec
b4+
b5+
b6+
y6+
b7+
y7+
m/z
200
400
600
800
0
1000
3000
IYKSDGIK+XL_L
KGTDIMYTGTLDCWRK+XL_S
B_b_ion
B_y_ion
y4+
b3+
b4+
y10++
y12++
y6+
b8+
y7+
y8+
prec
y9+
y10+
b12+
y11+
y12+
y13+
500
1000
1500
0
100
200
300
KGTDIMYTGTLDCWRK+XL_L
y4+
b4+
y10++
y5+
y6+
y14++
y12++
y11++
y13++
y7+
y8+
y9+
y10+
b12+
y11+
y12+
y13+
0
200
400
600
